# Changes in visceral adiposity, body composition, and cardiometabolic profiles following a supervised mixed exercise program in women with normal-weight obesity and obesity

**DOI:** 10.1186/s13102-026-01796-5

**Published:** 2026-06-11

**Authors:** Chuanzhi Wang, Ngai Choi, Jinzhao Li, Sumeng Xu, Zhide Liang, Xinfei Wen, Boyi Zhang, Wenyi Yang, Siman Lei, Sulin Cheng, Rui Duan, Xiuqiang Wang

**Affiliations:** 1 Lab of Regenerative Medicine in Sports Science, School of Physical Education and Sports Science, South China Normal University, Guangzhou, 510006 China; 2 Exercise Translational Medicine Center, Institute of Translational Medicine, Shanghai Jiao Tong University, Shanghai, 200240 China; 3 Key Laboratory of Systems Biomedicine (Ministry of Education), Shanghai Center for Systems Biomedicine, Shanghai Jiao Tong University, Shanghai, 200240 China; 4 Department of Physical Education, Shanghai Jiao Tong University, Shanghai, 200240 China; 5 Faculty of Health Sciences and Sports, Macao Polytechnic University, Rua de Gomes, Macao, China; 6 Department of Physical Education, Donghua University, Shanghai, China; 7 Institute of Cardiology and Sports Medicine, German Sport University Cologne, Cologne, Germany; 8 Faculty of Education, University of Macau, Taipa, Macau SAR 999078 China; 9 Center for Future Health and Intelligent Exercise, Global Institute of Future Technology, Shanghai Jiao Tong University, Shanghai, 200240 China; 10 National Center for Translational Medicine, Shanghai Jiao Tong University, Shanghai, 200240 China

**Keywords:** Normal-weight obesity, Visceral adipose tissue, Mixed exercise, Body composition, Cardiometabolic risk

## Abstract

**Objective:**

Normal-weight obesity (NWO) describes individuals with a normal body mass index (BMI) but elevated adiposity, potentially underestimating cardiometabolic risk. Mixed exercise may be particularly relevant for NWO, as it targets body composition, visceral adiposity, and cardiorespiratory fitness rather than weight loss alone. This study examined 12-week changes in DXA-derived body composition, regional adiposity, cardiorespiratory fitness, and cardiometabolic biomarkers in women with NWO and obesity (OB).

**Methods:**

Sixty-seven women aged 22–45 years who completed baseline and post-intervention assessments were included in the per-protocol analysis (NWO *n* = 32; OB *n* = 35). Participants were classified using predefined BMI and DXA-derived body fat criteria. Changes in outcomes were evaluated using 2 × 2 mixed-model repeated-measures ANOVA, with false discovery rate correction applied to within-group contrasts.

**Results:**

After 12 weeks, body weight and BMI decreased in the OB group but remained unchanged in the NWO group. Both groups demonstrated favorable changes in body composition. Total fat mass decreased from 24.68 ± 4.76 to 22.75 ± 3.84 kg in NWO and from 31.31 ± 3.91 to 29.46 ± 4.29 kg in OB, while total lean mass increased from 33.38 ± 4.00 to 34.70 ± 4.97 kg in NWO and from 39.04 ± 5.87 to 39.53 ± 5.90 kg in OB. Reductions were also observed across regional fat depots, including visceral adipose tissue (VAT), android fat, and gynoid fat. VAT decreased by − 58.2 g (95% CI, − 95.3 to − 21.1) in NWO and − 77.1 g (95% CI, − 109.4 to − 44.8) in OB, with parallel reductions in android and gynoid fat mass. The android-to-gynoid ratio showed minimal change. Cardiorespiratory fitness improved in both groups, with VO₂max increasing by 3.73 mL·kg^− 1^·min^− 1^ (95% CI, 0.75 to 6.72) in NWO and 3.03 mL·kg^− 1^·min^− 1^ (95% CI, 1.45 to 4.61) in OB. No significant group×time interactions were observed. Routine fasting cardiometabolic biomarkers showed limited or inconsistent changes after correction.

**Conclusions:**

A 12-week supervised mixed-exercise program improved adiposity and cardiorespiratory fitness in women with NWO and OB, independent of weight loss in NWO. No phenotype-specific differences were detected. These findings highlight the importance of assessing body composition beyond BMI, although causal inference is limited by the single-arm design.

**Trial registration:**

ChiCTR2100047173, registered on 9 June 2021, retrospectively registered.

**Supplementary Information:**

The online version contains supplementary material available at 10.1186/s13102-026-01796-5.

## Introduction

Obesity remains a major global health challenge and is closely associated with cardiometabolic disease, functional decline, and increased long-term morbidity [1, 2]. In clinical practice and public health surveillance, BMI is still the most commonly used index for identifying obesity. However, BMI is a crude proxy for metabolic risk, as it does not differentiate fat mass from fat-free mass and fails to capture regional adipose tissue distribution [3, 4]. This limitation is clinically important, because cardiometabolic risk is more closely related to visceral and ectopic fat accumulation than to body weight per se [5]. Visceral and central adiposity depots are biologically active and have been associated with adipose tissue dysfunction, low-grade inflammation, insulin resistance, dyslipidemia, and elevated cardiometabolic disease risk [5–8]. Thus, a weight-based definition of obesity may overlook individuals with a normal body weight but a metabolically unfavorable adiposity profile. Because BMI may fail to identify individuals with excess adiposity despite a normal body weight, increasing attention has been directed toward normal-weight obesity (NWO) [9]. NWO is generally characterized by a normal BMI with an elevated percent body fat [9–12]. In women, NWO is commonly defined as a BMI of 18.5–24.9 kg/m^2^ combined with percent body fat greater than 30% [9]. Although women with NWO are often overlooked by weight-centered screening, accumulating evidence indicates that this phenotype carries a higher cardiometabolic risk than normal-weight lean status, including greater risks of insulin resistance, dyslipidemia, hypertension, metabolic syndrome, and systemic inflammation [10, 11, 13]. This phenotype therefore poses a practical and scientific dilemma: a woman may be classified as normal weight by BMI while carrying an adiposity burden and distribution that warrants clinical attention.

Exercise and physical activity are central components of obesity management and are recommended to improve body composition, cardiometabolic health, and physical fitness [14, 15]. However, the intervention rationale for NWO differs from that for OB. In individuals with obesity, body-weight reduction is often treated as a visible marker of success. In women with NWO, the principal concern is not excess body weight per se, but the mismatch between a normal BMI and elevated adiposity. Therefore, meaningful changes in this phenotype may be more accurately captured by body composition and regional adiposity measures than by body weight alone. In this context, dual-energy X-ray absorptiometry (DXA) provides a more informative assessment framework, as it can quantify total fat mass, lean mass, visceral adipose tissue (VAT), android fat, and gynoid fat, thereby revealing adiposity patterns that BMI cannot detect [16].

Mixed-exercise training may be particularly relevant for this phenotype, because it combines complementary adaptive components. Aerobic exercise increases energy expenditure and improves cardiorespiratory fitness; resistance training supports muscle function and preserves lean mass; and high-intensity interval training elicits favorable changes in central and visceral adiposity [17, 18].

Previous randomized controlled trials, position statements, and meta-analyses have shown that aerobic, resistance, combined, and interval-based exercise programs are often accompanied by improvements in body composition, visceral adiposity, and cardiorespiratory fitness in populations with overweight or obesity [19, 20]. However, women with NWO have rarely been examined alongside women with OB under the same supervised mixed-exercise program. It therefore remains unclear whether these two phenotypes exhibit comparable or distinct changes in DXA-derived regional adiposity, body composition, cardiorespiratory fitness, and cardiometabolic biomarkers when evaluated within an identical outcome framework.

Our two published reports from the same registered parent research programme are directly relevant to the present manuscript. Cao et al. examined changes in fat oxidation and body composition following a 7-week combined exercise intervention in 56 mixed-sex completers [21], whereas Zhang et al. investigated sedentary behaviour patterns and related health outcomes in earlier recruitment waves [22].

Although these studies share overarching objectives, they address distinct aims and use separate datasets; accordingly, there is no sample overlap.

The present study extends this work by examining 12-week changes in DXA-derived body composition, regional adiposity, cardiorespiratory fitness, and cardiometabolic biomarkers in women with NWO and OB following a supervised mixed-exercise program.

## Methods

### Study design and ethical statement

This study was conducted as a cohort-specific, phenotype-stratified analysis nested within the registered parent project “Application of Exercise Intervention and Health Management in Sedentary Population” (Chinese Clinical Trial Registry, ChiCTR; www.chictr.org.cn; registration number: ChiCTR2100047173; retrospectively registered on 9 June 2021), originally designed as an approximately 3-month supervised exercise intervention. The present study employed a single-arm, quasi-experimental pre-post design, with standardized assessments performed at baseline (week 0) and after completion of the 12-week intervention (week 12).

The registered parent programme included multiple experimental phases and intervention cohorts. The present study is distinct in focusing on a separate 12-week, phenotype-stratified cohort of 67 women with normal-weight obesity or obesity, using DXA-derived regional adiposity and body composition, along with cardiometabolic biomarkers and cardiorespiratory fitness as outcomes. Participant-level identifiers were cross-checked against the previously published samples, confirming that no individuals were included in more than one analysis. Participants in the present analysis were stratified into two predefined body composition phenotypes: NWO and OB, according to baseline body mass index (BMI) and percent body fat (PBF). The primary aim was to compare phenotype-specific changes in DXA-derived body composition, regional adiposity, cardiorespiratory fitness, and cardiometabolic markers following the same supervised mixed-exercise program. The study protocol was approved by the Ethics Committee of Shanghai Jiao Tong University (approval no. B2020025I), and all participants provided written informed consent before enrollment and data collection.

### Participant recruitment and selection

Female participants were recruited between December 2020 and June 2022 through campus advertisements at Shanghai Jiao Tong University, social media platforms, and community health seminars. All participants completed baseline screening procedures, including lifestyle questionnaires, American College of Sports Medicine (ACSM) preparticipation health risk stratification, behavioral surveys, and physical examinations. Sedentary status was confirmed during screening using a standardized interview and was defined as spending most of the day sitting and not having engaged in regular exercise during the previous 3 months (fewer than two exercise sessions per week, with each session lasting less than 30 min) [22].

At baseline, participants were classified using predefined phenotypic criteria based on BMI and DXA-derived PBF. Women with a BMI of 18.5–24.9 kg/m² and a DXA-derived PBF > 30% were assigned to the NWO group, whereas women with a BMI ≥ 28.0 kg/m² and a DXA-derived PBF > 30% were assigned to the OB group. The NWO definition followed criteria commonly used in previous studies, in which women with a normal BMI but elevated DXA-derived PBF are classified as having normal-weight obesity. The OB definition was based on the Chinese diagnostic criteria for obesity [23, 24]. The InBody 720 was used solely for routine anthropometric and bioelectrical impedance measurements and was not used to classify participants into the NWO or OB phenotypes.

**Fig. 1:**
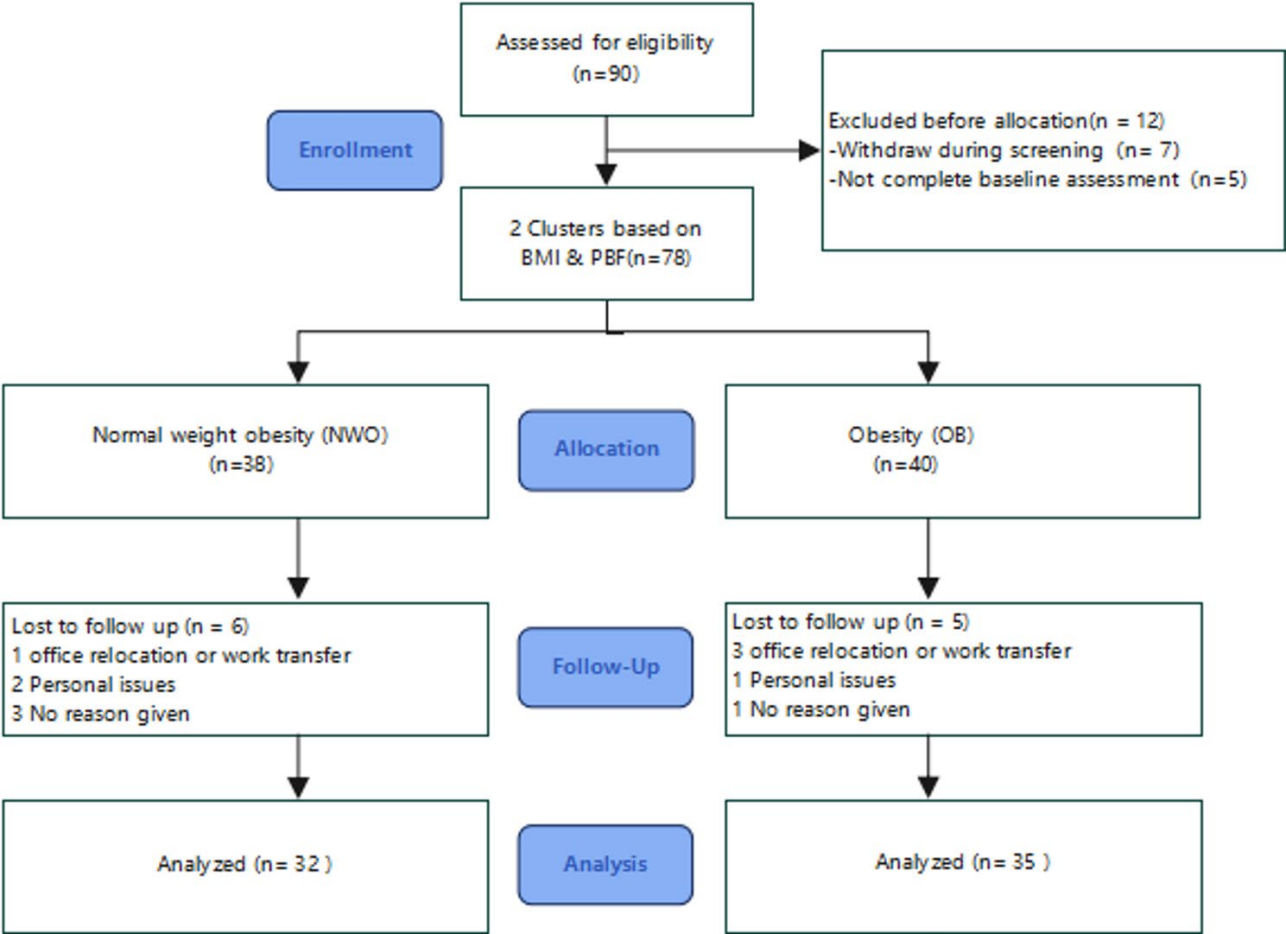
Study flow chart

Of the 90 women initially screened, 12 were excluded prior to intervention initiation due to eligibility criteria or withdrew before enrollment. The remaining 78 eligible participants commenced the intervention and were classified at baseline into the predefined phenotype-based subgroups. During the intervention and follow-up period, 11 participants were excluded from the final per-protocol analysis due to incomplete intervention exposure and/or missing week-12 outcome data. Reasons for discontinuation or loss to follow-up included office relocation or work transfer (*n* = 4), personal reasons (*n* = 3), and no stated reason (*n* = 4). Specifically, six participants in the NWO group were not included in the final analysis: one due to office relocation or work transfer, two due to personal reasons, and three with no reason given. Five participants in the OB group were not included in the final analysis: three due to office relocation or work transfer, one due to personal reasons, and one with no reason given. Attendance was recorded at each session, and adherence was calculated as the number of completed sessions relative to the prescribed 36 sessions. Accordingly, 67 women completed both assessment visits and were included in the final per-protocol analysis: 32 in the NWO group and 35 in the OB group. The participant flow and reasons for exclusion are presented in Fig. 1.

Inclusion criteria were: (1) women aged 22–45 years; (2) confirmed sedentary status; (3) fulfillment of the predefined baseline phenotypic criteria for either NWO or OB; and (4) no cardiovascular, metabolic, or musculoskeletal contraindications to moderate-to-vigorous exercise.

Exclusion criteria were: (1) class III obesity (BMI > 40 kg/m^2^); (2) severe cardiovascular disease; (3) recent musculoskeletal injury; (4) pregnancy or lactation; (5) participation in structured weight-loss programs or body-weight instability (> 5% change) within the previous 6 months; and (6) use of medications or supplements known to affect lipid metabolism, appetite, or body weight.

### Supervised mixed-exercise program

The supervised mixed-exercise protocol was based on the established intervention framework used in the registered parent programme and was informed by previously published combined-exercise protocols from the same research programme [22]. ACSM’s preparticipation health screening recommendations from the 9th edition of ACSM’s Guidelines for Exercise Testing and Prescription were cited as the formal reference for preparticipation health risk stratification and safety screening [25]. General exercise-intensity monitoring, progressive load adjustment, and safety supervision were implemented as part of the established supervised intervention framework. Briefly, participants completed a 12-week supervised mixed-exercise training program performed three times per week on non-consecutive days. Each session lasted approximately 70–75 min and was delivered individually under the supervision of certified exercise physiologists to ensure safety, adherence, and proper technique.

**Fig. 2:**
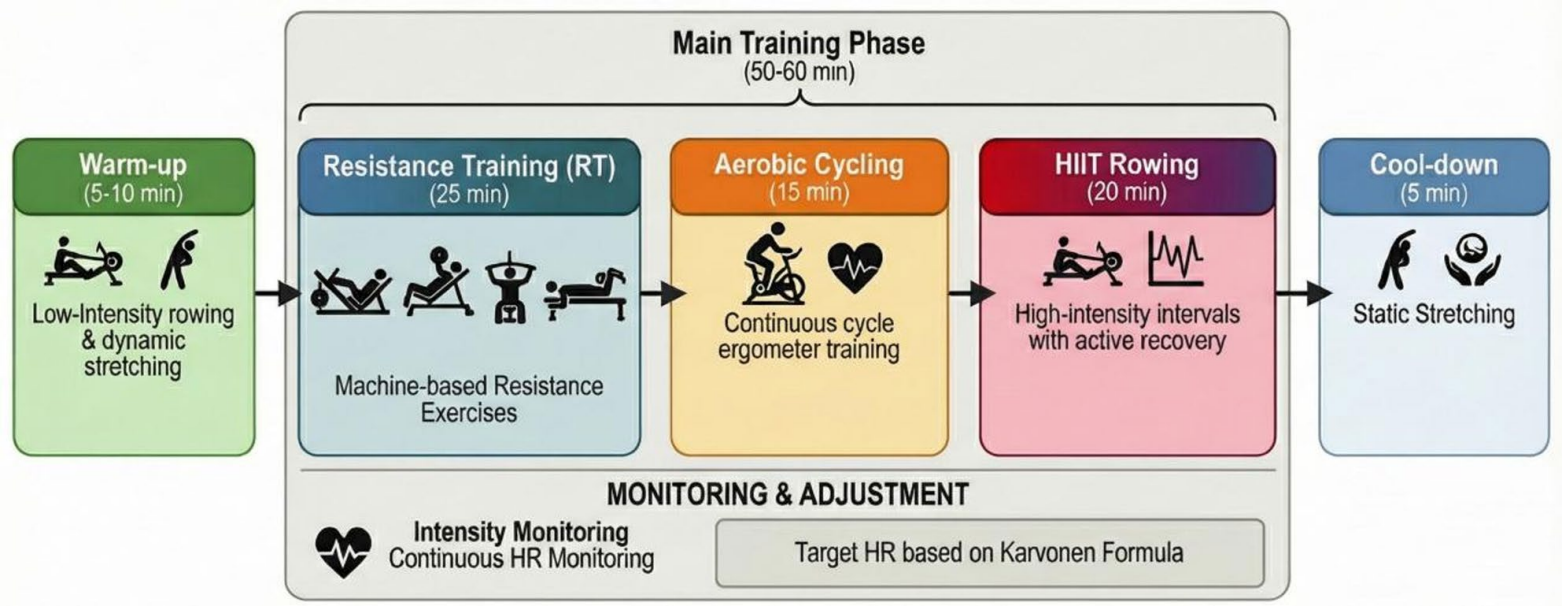
Structure of the 12-week supervised mixed-exercise intervention, comprising warm-up, machine-based resistance training (seated leg press, incline chest press, lat pulldown, and prone leg curl), continuous aerobic cycling, HIIT rowing, and cool-down

The session structure was as follows:

1. Warm-up (5–10 min): Low-intensity rowing (DOMYOS 500, Decathlon, France) combined with dynamic whole-body stretching at <40% heart rate reserve (HRR).
2. Main training phase (60 min):
  Resistance training (25 min): As illustrated in Figure 2, the first component of the main training phase, participants performed a fixed sequence of four machine-based resistance exercises targeting the major upper- and lower-body muscle groups: seated leg press, incline chest press, lat pulldown, and prone leg curl. The resistance-training block was performed in a circuit-style pyramidal format. Specifically, one set of each exercise was completed sequentially at each load level before progressing to the next load level, such that the entire circuit was repeated four times at 50%, 60%, 70%, and 80% of one-repetition maximum (1RM). For each set, participants completed 8–12 repetitions with a controlled movement tempo (1–2 s concentric and 2–3 s eccentric). A 1-min rest interval was provided between completed circuit rounds, with brief transition time allowed between exercises as needed. Training loads were re-evaluated every 4 weeks based on repeated 1RM assessments and adjusted to maintain progressive overload. Exercise loads were calculated from the reassessed 1RM values and rounded to the nearest 5 kg according to the smallest available machine-load increment.
  Continuous cycle ergometry (15 min): Performed on a Monark LC7 ergometer (Monark Exercise AB, Vansbro, Sweden) at 40%–70% of peak power output determined during baseline cardiorespiratory fitness testing.
  HIIT rowing (20 min): Performed on a DOMYOS 500 magnetic-resistance rowing machine (Decathlon, France) as five cycles of 1 min at ~90% of individual maximal load, interspersed with 3 min of active recovery at ~40%. Stroke rate was maintained at ~26 strokes/min. Heart rate was continuously monitored using a Polar H10 chest strap (Polar Electro, Finland) to verify intensity and ensure safety.
3. Cool-down (5 min): Static stretching of the major muscle groups, including the quadriceps, hamstrings, back, and pectorals.

### Progression and monitoring

Progressive overload was implemented every 4 weeks based on repeated 1RM reassessments, together with interim adjustments of aerobic and interval training loads. These interim reassessments were used solely for exercise prescription adjustment and were not treated as formal study outcome assessments. No dietary intervention was implemented during the study; participants were instructed to maintain their habitual dietary patterns throughout the 12-week intervention.

### Outcomes and measurement instruments

All assessments were performed at baseline and upon completion of the 12-week intervention. Participants were instructed to fast overnight for 10–12 h, void prior to testing, and refrain from vigorous exercise, alcohol, and caffeine for 24 h before each assessment session.

The primary outcomes were changes in DXA-derived adiposity measures, including visceral adipose tissue (VAT) mass, total fat mass, and regional fat mass (android and gynoid). Changes in lean mass were also assessed to characterize body composition responses. Secondary outcomes included maximal oxygen uptake (VO₂max), resting blood pressure, and fasting cardiometabolic biomarkers.

### Anthropometry and body composition

Height was measured to the nearest 0.1 cm using a wall-mounted stadiometer. Body weight and bioimpedance-derived indices were measured using a multi-frequency segmental bioelectrical impedance analyzer (InBody 720; Biospace Co., Ltd., Seoul, Korea). Measurements were conducted with participants standing barefoot on the foot electrodes while holding the hand electrodes, in accordance with the manufacturer’s instructions. The device employs an eight-point tactile electrode system, incorporating contacts at the thumbs, palms, forefeet, and heels, and performs direct segmental multi-frequency impedance analysis. All assessments were carried out following a 10–12-hour overnight fast, and participants were instructed to void their bladder prior to measurement.

Whole-body and regional body composition were quantified using dual-energy X-ray absorptiometry (DXA; Lunar Prodigy, GE Healthcare, Shanghai, China; enCORE software version 12.2). The measured outcomes included total fat mass, lean mass, percent body fat, VAT mass, android fat mass, and gynoid fat mass. All scans were performed and analyzed by the same certified technician. Daily phantom calibration was conducted, and the coefficient of variation was maintained at < 1%.

### Resting blood pressure

After 10 min of supine rest in a quiet, temperature-controlled room, resting blood pressure was measured twice at the left brachial artery using a validated automated oscillometric device (BP-203RPE; Omron, Kyoto, Japan). The mean of the two measurements was used for analysis.

### Cardiorespiratory fitness

VO_2_max was determined during a graded exercise test on a cycle ergometer (Monark LC7; Monark Exercise AB, Vansbro, Sweden). Participants were instructed to maintain a pedal cadence of 60 rpm throughout the test. The protocol began at 50 W and was incremented by 20 W every 3 min until volitional exhaustion. VO_2_max was considered to be achieved when at least two of the following criteria were met: (1) heart rate ≥ 90% of the age-predicted maximum (220 − age); (2) rating of perceived exertion ≥ 18 on the Borg scale; (3) respiratory exchange ratio ≥ 1.10; or (4) a plateau in oxygen uptake despite increasing workload. Expired gases were analyzed breath-by-breath using a calibrated metabolic cart (Cortex MetaLyzer 3B; Cortex Biophysik GmbH, Leipzig, Germany), and heart rate was continuously monitored using a Polar H10 device.

The ventilatory anaerobic threshold was not included as an outcome because the original study protocol was designed to evaluate maximal aerobic capacity rather than submaximal ventilatory thresholds. In addition, ventilatory anaerobic threshold determination was not prespecified in the registered protocol and was not independently adjudicated using standardized dual-review procedures. Therefore, VO_2_max was retained as the cardiorespiratory fitness outcome in the present analysis.

VO_2_max was reported in three formats: relative to total body mass (mL·kg^− 1^·min^− 1^), in absolute terms (L·min^− 1^), and relative to lean body mass (mL·kg LBM^− 1^·min^− 1^). Absolute VO_2_max was calculated as VO_2_max expressed relative to body mass multiplied by body weight and divided by 1000. VO_2_max relative to lean body mass was calculated as absolute VO_2_max multiplied by 1000 and divided by DXA-derived total lean mass.

### Blood sampling and biochemical analyses

Following a 10–12 h overnight fast, venous blood samples (~ 15 mL) were drawn from the antecubital vein between 07:00 and 09:00. Plasma and serum were separated by centrifugation (3000 rpm for 10 min at 4 °C) and stored at − 80 °C until batch analysis. Fasting glucose, triglycerides, total cholesterol, low-density lipoprotein cholesterol (LDL-C), high-density lipoprotein cholesterol (HDL-C), and free fatty acids (FFAs) were measured using automated enzymatic methods on an AU680 automated biochemical analyzer (Beckman Coulter, Brea, CA, USA). Fasting insulin was measured using an electrochemiluminescence immunoassay on a Cobas e602 analyzer (Roche, Basel, Switzerland). Glycated hemoglobin (HbA1c) was measured using an H50 glycated hemoglobin analyzer (Mindray, Shenzhen, China).

Insulin resistance was estimated using the Homeostatic Model Assessment of Insulin Resistance (HOMA-IR), calculated as:

HOMA-IR = [fasting glucose (mmol/L) × fasting insulin (µU/mL)] / 22.5.

When fasting insulin was reported in pmol/L, values were converted to µU/mL prior to calculation using the conversion factor 1 µU/mL = 6.945 pmol/L. All biochemical assays were performed in a certified clinical laboratory in accordance with standard operating procedures.

### Statistical analysis

The sample size was estimated using G*Power (version 3.1) for a repeated-measures ANOVA with a within–between interaction, assuming an effect size of f = 0.40, a two-sided α of 0.05, statistical power of 0.80, two groups, two repeated measurements, and a correlation of 0.50 among repeated measures. The required total sample size was 28 (14 participants per subgroup), which was exceeded in the present study.

All analyses were performed using R software (version 4.4.1; R Foundation for Statistical Computing, Vienna, Austria) on a per-protocol basis, including only participants with complete baseline and week-12 data. The main R packages used for data management, statistical testing, repeated-measures modelling, multiple-comparison adjustment, effect-size estimation, and visualization are listed with package versions and formal citations in Supplementary Table S2. Continuous variables are presented as mean ± standard deviation (SD). Normality was assessed using the Shapiro–Wilk test together with visual inspection of Q–Q plots. For variables with markedly skewed distributions, natural-log transformation was applied prior to parametric modeling when this improved model assumptions. Descriptive statistics are reported on the original scale to preserve clinical interpretability.

Baseline between-group differences were assessed using independent-samples t tests for normally distributed variables. For variables that remained non-normally distributed and unsuitable for parametric testing, Mann–Whitney U tests were used for baseline comparisons only.

Intervention-related changes were evaluated using a 2 × 2 mixed-model repeated-measures ANOVA, with time (baseline vs. week 12) as the within-subject factor and group (NWO vs. OB) as the between-subject factor. Main effects of time and group, as well as the group × time interaction, were examined for each outcome. When log transformation was applied, transformed values were used for inferential testing, while raw-scale values were retained in the tables for interpretability. Because only two repeated measurements were included, no sphericity correction was required.

Within-group pre–post contrasts were reported as supplementary analyses to characterize changes within each phenotype group. These contrasts were not used to infer between-group differences in intervention response. Between-group differences in change from baseline to week 12 were interpreted on the basis of the group × time interaction. To account for multiple supplementary within-group contrasts, the Benjamini–Hochberg false discovery rate (FDR) procedure was applied within predefined outcome families, given the exploratory nature of these contrasts and the correlations among related outcomes. For Table 2, anthropometric and body composition outcomes were treated as a single family. For Table 3, physiological and metabolic outcomes were treated as one family, while VO_2_max indices were treated as a separate cardiorespiratory fitness outcome family. Both nominal and FDR-adjusted P values are reported for within-group contrasts.

Mean differences (MDs) with 95% confidence intervals (CIs) were calculated to summarize changes from baseline to week 12. Effect sizes for within-group changes were expressed as standardized mean changes, calculated as the mean change divided by the baseline SD. All statistical tests were two-sided, and statistical significance was set at *P* < 0.05.

To account for baseline differences between the NWO and OB groups, baseline-adjusted ANCOVA models were additionally applied to post-intervention outcomes, with the corresponding baseline value included as a covariate. Adjusted mean differences were calculated as OB minus NWO, while outcomes that were log-transformed are presented as OB-to-NWO ratios.

## Results

### Baseline characteristics

Baseline characteristics of the 67 women included in the per-protocol analysis are summarized in Table 1. Compared with the NWO group, the OB group exhibited significantly higher body weight, BMI, total fat mass, total lean mass, VAT mass, android fat mass, and gynoid fat mass. By definition, women in the NWO group had a normal BMI but elevated percent body fat, with a mean percent body fat of 42.50%, VAT mass of 481 ± 126 g, and android fat mass of 1779 ± 334 g. Thus, the NWO group met the predefined phenotypic criteria of a normal BMI combined with elevated percent body fat and measurable regional adiposity.

With respect to cardiometabolic and functional characteristics, the OB group showed higher diastolic blood pressure (DBP) and HOMA-IR than the NWO group, while fasting glucose showed a borderline between-group difference. Lipid profiles, including triglycerides, total cholesterol, HDL-C, and LDL-C, did not differ significantly between groups. VO₂max was significantly lower in the OB group than in the NWO group. Overall, these baseline findings indicate that the two phenotypes differed primarily in overall body size, regional adiposity, insulin resistance, and cardiorespiratory fitness, whereas several routine lipid markers were broadly comparable at baseline.

Participant retention and attrition-related baseline comparisons are presented in Supplementary Table S1. Of the 78 participants who commenced the intervention, 67 completed both baseline and week-12 assessments and were included in the final per-protocol analysis. The attrition rate was 15.8% in the NWO group and 12.5% in the OB group, with no significant between-group difference. Most baseline characteristics were comparable between completers and non-completers, although non-completers were taller and had lower triglyceride levels at baseline. Accordingly, attrition was not clearly phenotype-specific, although residual attrition-related imbalance cannot be entirely excluded.

**Table 1:** Baseline characteristics of participants included in the per-protocol analysis

Variable	NWO Group (*n* = 32)	OB Group (*n* = 35)	*P*
Age (years)	39.86 ± 8.73	40.50 ± 7.27	0.747
Height (cm)	160.13 ± 5.41	155.94 ± 5.31	0.003
Weight (kg)	58.06 ± 4.75	70.35 ± 8.63	< 0.001
BMI (kg/m²)	22.64 ± 1.01	28.93 ± 2.88	< 0.001
Systolic BP (mmHg)	122.35 ± 24.21	131.49 ± 20.46	0.147
Diastolic BP (mmHg)	66.40 ± 10.95	73.50 ± 12.10	0.031
Body fat percentage (%)	42.50 ± 6.49	44.50 ± 4.97	0.220
Total fat mass (kg)	24.68 ± 4.76	31.31 ± 3.91	< 0.001
Total lean mass (kg)	33.38 ± 4.00	39.04 ± 5.87	< 0.001
Visceral fat mass (g)	481 ± 126	686 ± 230	< 0.001
Android fat mass (g)	1779 ± 334	2391 ± 531	< 0.001
Gynoid fat mass (g)	3919 ± 1004	4814 ± 826	< 0.001
Android/gynoid ratio	0.47 ± 0.13	0.50 ± 0.08	0.444
Fasting glucose (mmol/L)	5.16 ± 0.66	5.51 ± 0.67	0.052
HbA1c (%)	5.50 ± 0.64	5.65 ± 0.38	0.323
TG† (mmol/L)	1.04 ± 0.65	1.15 ± 0.57	0.517
TC (mmol/L)	4.99 ± 0.97	5.22 ± 1.32	0.446
HDL-C (mmol/L)	1.55 ± 0.31	1.47 ± 0.40	0.351
LDL-C (mmol/L)	3.17 ± 0.98	3.26 ± 0.71	0.696
HOMA-IR†	2.10 ± 1.21	2.96 ± 2.05	0.040
Insulin† (pmol/L)	62.80 ± 33.00	82.0 ± 51.4	0.075
VO_2_max (ml/kg/min)	29.40 ± 4.70	25.14 ± 3.63	0.005

Data are presented as mean ± SD. Baseline differences between the NWO and OB groups were assessed using independent-samples t tests unless otherwise indicated. † indicates that the P value was calculated using the Mann–Whitney U test because the variable was not suitable for parametric testing. Bold values indicate statistical significance at *P* < 0.05. *NWO* normal-weight obesity, *OB* obesity, *BMI* body mass index, *BP* blood pressure, *HDL-C* high-density lipoprotein cholesterol, *LDL-C* low-density lipoprotein cholesterol, *HOMA-IR* homeostatic model assessment for insulin resistance, VO_2_max maximal oxygen uptake

### Changes in body composition and regional adiposity

**Fig. 3:**
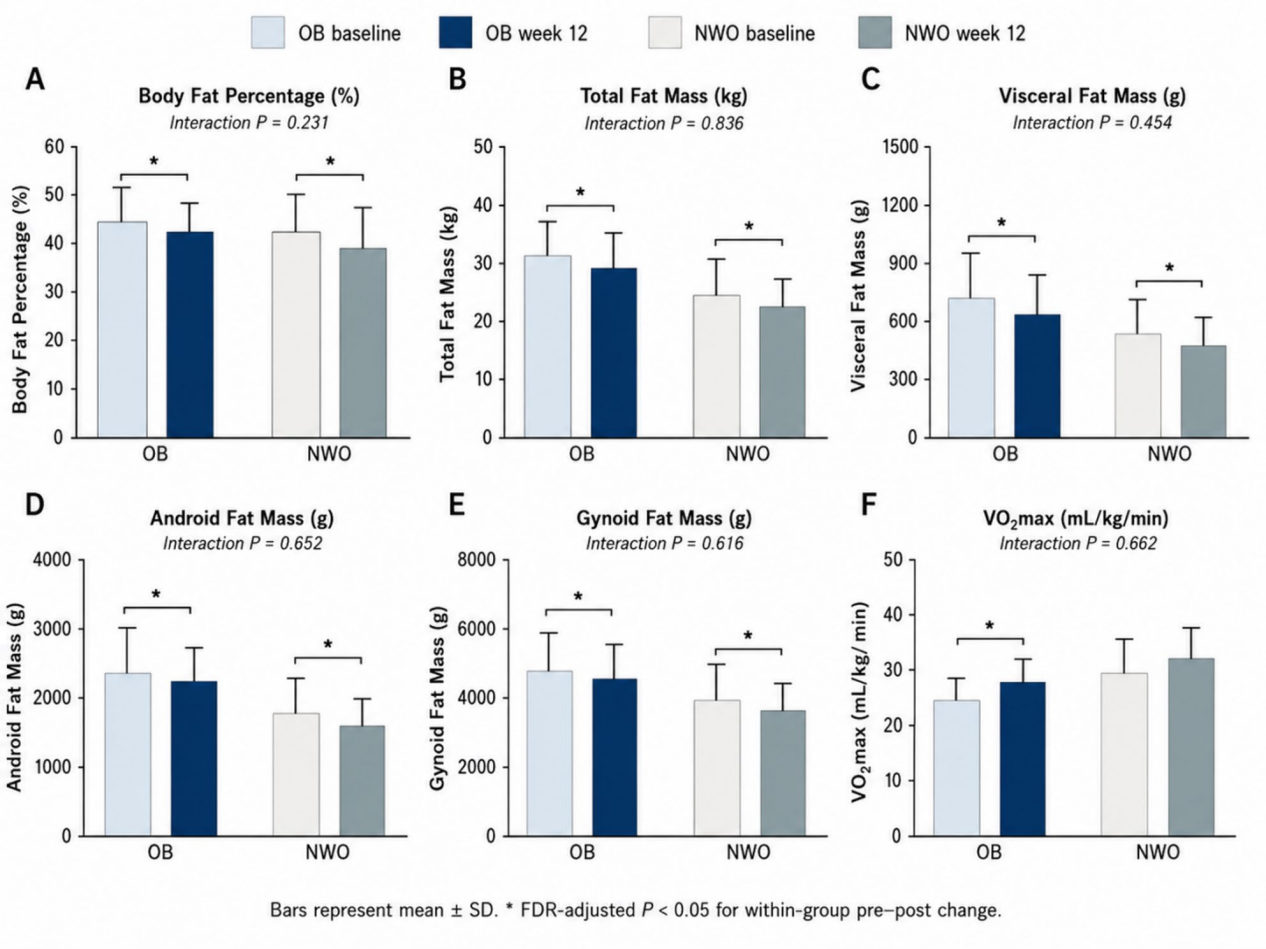
Changes in body composition, regional adiposity, and cardiorespiratory fitness from baseline to week 12 in women with obesity and normal-weight obesity. Panels show changes in (**A**) body fat percentage, **B** total fat mass, **C** visceral fat mass, **D** android fat mass, **E** gynoid fat mass, and **F** VO₂max. Bars represent mean ± SD. Interaction P values indicate the group × time effect derived from the 2 × 2 mixed repeated-measures ANOVA. Horizontal brackets indicate within-group pre–post contrasts. * indicates FDR-adjusted *P* < 0.05 for within-group comparisons

From baseline to week 12, significant main effects of time were observed for several anthropometric and body composition outcomes, indicating overall changes across both phenotypes (Table 2; Fig. 3). The OB group exhibited reductions in body weight and BMI that remained significant after FDR correction, whereas these variables remained largely unchanged in the NWO group. However, no significant group × time interactions were detected for body weight or BMI, indicating that the magnitude of change did not differ significantly between phenotypes.

Adiposity-related outcomes showed more consistent changes than body weight. In the NWO group, percent body fat, total fat mass, VAT mass, android fat mass, and gynoid fat mass all decreased from baseline to week 12, and these changes remained significant after FDR correction. Comparable reductions in adiposity-related outcomes were also observed in the OB group. These findings suggest that DXA-derived body composition and regional adiposity measures captured changes that were not fully reflected by body weight, particularly in women with NWO.

Lean mass showed a nominal increase in the NWO group, but this change did not remain statistically significant after FDR correction; this finding should therefore be interpreted with caution. The android-to-gynoid ratio (A/G ratio) remained unchanged in both groups, indicating that reductions in regional fat mass were not accompanied by a statistically significant shift in the relative fat distribution between central and gluteofemoral depots. Across body composition outcomes, group × time interactions were not significant, providing no clear evidence that the magnitude of change differed between the NWO and OB groups.

Given the substantial baseline differences in body size and adiposity between the NWO and OB groups, post-intervention between-group comparisons were further examined using baseline-adjusted ANCOVA models. After adjusting for baseline values, most differences in body composition and regional adiposity were attenuated. Although total fat mass showed a nominal adjusted difference between OB and NWO, this did not remain statistically significant after false discovery rate (FDR) correction. Similar patterns were observed for android fat mass, gynoid fat mass, visceral adipose tissue (VAT) mass, and the android-to-gynoid fat ratio. In contrast, VO_2_max remained significantly lower in the OB group compared with the NWO group after baseline adjustment and FDR correction. These results are presented in Supplementary Table S3.

**Table 2:** Changes in anthropometric and body composition outcomes from baseline to week 12

Variable	Group	Baseline	Week 12	Change (95% CI)	Pwithin	Pwithin, FDR	d	Ptime	Pgroup	Pgroup × time
Weight (kg)	NWO	58.06 ± 4.75	57.45 ± 5.29	−0.62 (− 1.44, 0.20)	0.154	0.173	−0.13	< 0.001	< 0.001	0.209
	OB	70.35 ± 8.63	68.99 ± 8.83	−1.35 (− 2.13, − 0.57)	0.002	0.004	−0.16			
BMI (kg/m²)	NWO	22.64 ± 1.01	22.32 ± 1.32	−0.30 (− 0.69, 0.09)	0.151	0.173	−0.29	0.002	< 0.001	0.465
	OB	28.93 ± 2.88	28.45 ± 2.99	−0.48 (− 0.75, − 0.20)	0.002	0.004	−0.17			
Body fat percentage (%)	NWO	42.50 ± 6.50	39.60 ± 5.60	−2.93 (− 4.94, − 0.92)	0.009	0.016	−0.45	< 0.001	0.031	0.231
	OB	44.50 ± 5.00	42.70 ± 3.30	−1.75 (− 2.95, − 0.55)	0.007	0.011	−0.35			
Total fat mass (kg)	NWO	24.68 ± 4.76	22.75 ± 3.84	−1.93 (− 3.17, − 0.69)	0.005	0.016	−0.41	< 0.001	< 0.001	0.836
	OB	31.31 ± 3.91	29.46 ± 4.29	−1.85 (− 2.55, − 1.15)	0.001	0.003	−0.47			
Lean mass (kg)	NWO	33.38 ± 4.00	34.70 ± 4.97	1.32 (0.06, 2.58)	0.045	0.067	0.33	0.014	< 0.001	0.234
	OB	39.04 ± 5.87	39.53 ± 5.90	0.49 (− 0.24, 1.22)	0.180	0.202	0.08			
Visceral fat mass (g)	NWO	481 ± 126	423 ± 116	−58.2 (− 95.3, − 21.1)	0.006	0.016	−0.46	< 0.001	< 0.001	0.454
	OB	686 ± 230	608 ± 190	−77.1 (− 109.4, − 44.8)	0.001	0.003	−0.34			
Android fat mass (g)	NWO	1779 ± 334	1617 ± 379	−162 (− 265, − 58)	0.006	0.016	−0.49	< 0.001	< 0.001	0.652
	OB	2391 ± 531	2258 ± 495	−132 (− 207, − 58)	0.001	0.003	−0.25			
Gynoid fat mass (g)	NWO	3919 ± 1004	3671 ± 977	−248 (− 417, − 79)	0.009	0.016	−0.25	< 0.001	< 0.001	0.616
	OB	4814 ± 826	4622 ± 734	−192 (− 332, − 52)	0.010	0.013	−0.23			
Android/gynoid ratio	NWO	0.47 ± 0.13	0.46 ± 0.14	−0.01 (− 0.03, 0.01)	0.332	0.332	−0.08	0.075	0.270	0.869
	OB	0.50 ± 0.08	0.49 ± 0.08	−0.01 (− 0.02, 0.00)	0.221	0.221	−0.11			

Data are presented as mean ± SD unless otherwise specified. Change (95% CI) represents the unadjusted mean difference from baseline to week 12 with its 95% confidence interval. Pwithin denotes the nominal *P* value for supplementary within-group pre–post contrasts. Pwithin, FDR denotes the Benjamini–Hochberg false discovery rate-adjusted P value for these within-group contrasts. The FDR adjustment was performed separately within each phenotype group across the anthropometric and body composition outcomes shown in this table. These within-group contrasts were reported for descriptive purposes and were not used to infer between-group differences in intervention response. Ptime, Pgroup, and Pgroup × time denote the main effect of time, the main effect of group, and the group × time interaction derived from the 2 × 2 mixed repeated-measures ANOVA, respectively. These model-based P values are reported once for each outcome. Between-group differences in changes from baseline to week 12 were interpreted according to the group × time interaction. d represents the standardized mean change, calculated as the mean change divided by the baseline SD. Bold values indicate statistical significance after FDR adjustment where applicable (*P* < 0.05). No variables in this table required natural-log transformation for inferential testing. *NWO* normal-weight obesity, *OB* obesity, *BMI* body mass index, *CI* confidence interval, *FDR* false discovery rate

### Changes in physiological, metabolic, and cardiorespiratory fitness outcomes

Physiological, metabolic, and cardiorespiratory fitness outcomes are summarized in Table 3. Overall, routine cardiometabolic biomarkers showed more limited and less consistent responses compared with body composition outcomes. Significant main effects of time were observed for systolic blood pressure (SBP), diastolic blood pressure (DBP), LDL-C, and the three VO₂max indices, whereas group × time interactions were not significant for any of these outcomes.

Among lipid outcomes, LDL-C showed a nominal reduction in both groups. After FDR correction, this reduction remained significant in the OB group but not in the NWO group. The remaining lipid markers, including triglycerides, total cholesterol, and HDL-C, did not show consistent within-group changes after FDR correction. Markers of glucose homeostasis, including fasting glucose, HbA1c, fasting insulin, and HOMA-IR, showed no significant FDR-adjusted within-group changes in either group.

Cardiorespiratory fitness was expressed as VO₂max relative to body mass, absolute VO₂max, and VO₂max relative to lean body mass. Significant main effects of time were observed for all three indices, indicating overall improvements from baseline to week 12. VO₂max relative to body mass increased from 29.40 ± 4.72 to 33.13 ± 3.96 mL·kg⁻¹·min⁻¹ in the NWO group and from 25.14 ± 3.63 to 28.17 ± 3.97 mL·kg⁻¹·min⁻¹ in the OB group. Comparable directional increases were observed for absolute VO₂max and lean-mass-normalized VO₂max. No significant group × time interactions were detected for any of the VO₂max indices, indicating that the magnitude of change did not differ significantly between phenotypes.

**Table 3:** Changes in physiological, metabolic, and fitness profiles

Variable	Group	Baseline	Week 12	Change (95% CI)	Pwithin	Pwithin, FDR	d	Ptime	Pgroup	Pgroup × time
SBP (mmHg)	NWO	122.35 ± 24.21	117.10 ± 20.10	−5.24 (− 10.98, 0.49)	0.088	0.440	−0.22	0.037	0.029	0.443
	OB	131.49 ± 20.46	129.60 ± 18.80	−2.38 (− 6.82, 2.07)	0.301	0.376	−0.12			
DBP (mmHg)	NWO	66.40 ± 11.00	63.60 ± 6.80	−2.80 (− 6.34, 0.75)	0.138	0.460	−0.26	0.003	0.004	0.604
	OB	73.50 ± 12.10	69.40 ± 9.00	−4.16 (− 7.17, − 1.15)	0.010	0.050	−0.34			
Glucose (mmol/L)	NWO	5.16 ± 0.66	5.45 ± 1.12	0.29 (− 0.22, 0.80)	0.280	0.560	0.44	0.389	0.218	0.221
	OB	5.51 ± 0.67	5.45 ± 0.55	−0.06 (− 0.23, 0.11)	0.524	0.582	−0.08			
HbA1c (%)	NWO	5.50 ± 0.64	5.58 ± 0.59	0.09 (− 0.05, 0.22)	0.231	0.560	0.13	0.371	0.377	0.312
	OB	5.65 ± 0.38	5.64 ± 0.37	−0.01 (− 0.13, 0.11)	0.880	0.880	−0.02			
TG† (mmol/L)	NWO	1.04 ± 0.65	1.02 ± 0.43	−0.02 (− 0.32, 0.28)	0.892	0.892	−0.03	0.679	0.140	0.510
	OB	1.15 ± 0.57	1.25 ± 0.64	0.09 (− 0.07, 0.26)	0.272	0.376	0.17			
TC (mmol/L)	NWO	4.99 ± 0.97	4.87 ± 0.83	−0.12 (− 0.44, 0.19)	0.450	0.750	−0.13	0.153	0.415	0.696
	OB	5.22 ± 1.32	5.00 ± 0.90	−0.22 (− 0.58, 0.14)	0.242	0.376	−0.17			
HDL-C (mmol/L)	NWO	1.55 ± 0.31	1.56 ± 0.25	0.01 (− 0.10, 0.12)	0.884	0.892	0.03	0.320	0.404	0.499
	OB	1.47 ± 0.40	1.52 ± 0.33	0.06 (− 0.03, 0.15)	0.221	0.376	0.14			
LDL-C (mmol/L)	NWO	3.17 ± 0.98	2.85 ± 0.81	−0.32 (− 0.58, − 0.05)	0.029	0.290	−0.32	< 0.001	0.475	0.603
	OB	3.26 ± 0.71	3.03 ± 0.76	−0.24 (− 0.38, − 0.09)	0.003	0.030	−0.33			
HOMA-IR†	NWO	2.10 ± 1.21	2.32 ± 1.76	0.21 (− 0.71, 1.14)	0.657	0.892	0.18	0.317	0.026	0.853
	OB	2.96 ± 2.05	3.27 ± 2.52	0.32 (− 0.22, 0.85)	0.257	0.376	0.15			
Insulin† (pmol/L)	NWO	62.80 ± 33.00	64.30 ± 35.00	1.48 (− 19.7, 22.7)	0.892	0.892	0.04	0.398	0.020	0.561
	OB	82.00 ± 51.40	90.90 ± 61.20	8.90 (− 4.02, 21.8)	0.184	0.376	0.17			
VO_2_max (mL/kg/min)	NWO	29.40 ± 4.72	33.13 ± 3.96	3.73 (0.75, 6.72)	0.018	0.050	0.79	< 0.001	< 0.001	0.662
	OB	25.14 ± 3.63	28.17 ± 3.97	3.03 (1.45, 4.61)	< 0.01	< 0.01	0.83			
VO_2_max (L/min)	NWO	1.74 ± 0.31	1.92 ± 0.26	0.17 (-0.01, 0.36)	0.058	0.058	0.57	< 0.001	0.897	0.949
	OB	1.75 ± 0.25	1.93 ± 0.39	0.18 (0.06, 0.30)	0.005	0.005	0.74			
VO_2_max (mL/kg LBM/min)	NWO	47.0 ± 8.3	52.1 ± 8.3	5.1 (0.5, 9.8)	0.034	0.050	0.62	< 0.001	0.040	0.784
	OB	42.7 ± 6.8	47.2 ± 6.8	4.5 (2.1, 6.8)	< 0.001	< 0.001	0.65			

Data are presented as mean ± SD unless otherwise specified. Change (95% CI) represents the unadjusted mean difference from baseline to week 12 with its 95% confidence interval. † indicates that inferential *P* values were calculated using natural-log-transformed values, whereas raw-scale means, SDs, and mean changes are presented for interpretability. Pwithin denotes the nominal P value for supplementary within-group pre–post contrasts. Pwithin, FDR denotes the Benjamini–Hochberg false discovery rate-adjusted P value for these within-group contrasts. The FDR adjustment was performed separately within each phenotype group. Physiological and metabolic outcomes were treated as one outcome family, whereas VO₂max indices were treated as a separate cardiorespiratory fitness outcome family. These within-group contrasts were reported for descriptive purposes and were not used to infer between-group differences in intervention response. Ptime, Pgroup, and Pgroup×time denote the main effect of time, the main effect of group, and the group × time interaction derived from the 2 × 2 mixed repeated-measures ANOVA, respectively. These model-based P values are reported once for each outcome. Between-group differences in changes from baseline to week 12 were interpreted according to the group × time interaction. d represents the standardized mean change, calculated as the mean change divided by the baseline SD. Bold values indicate statistical significance after FDR adjustment where applicable (*P* < 0.05). *NWO* normal-weight obesity, *OB* obesity, *BMI* body mass index, *CI* confidence interval, *FDR* false discovery rate

## Discussion

In this phenotype-stratified analysis, the most pronounced changes from baseline to week 12 were observed in DXA-derived body composition, regional adiposity, and cardiorespiratory fitness. Women with NWO exhibited reductions in percent body fat, total fat mass, VAT mass, android fat mass, and gynoid fat mass, while body weight and BMI changed only marginally. Women with OB showed similar directional changes in adiposity-related outcomes, together with modest reductions in body weight and BMI. These findings suggest that the principal signal of change during the 12-week supervised mixed-exercise program was more clearly captured by body composition and fitness outcomes than by body weight or routine fasting metabolic biomarkers.

To aid interpretation, it is important to distinguish longitudinal changes from post-intervention differences between phenotypes. Given the substantial baseline differences in adiposity between the NWO and OB groups, baseline-adjusted analyses were conducted to assess whether residual differences reflected phenotype-specific exercise responses or initial disparities. After adjustment, most between-group differences in DXA-derived body composition and regional adiposity, including total fat mass, visceral adipose tissue, android and gynoid fat mass, and the android-to-gynoid) ratio, were attenuated and no longer significant after false discovery rate (FDR) correction. These findings suggest that residual differences after 12 weeks were largely driven by baseline characteristics rather than true differences in fat-loss responsiveness. Accordingly, both NWO and OB groups appear to exhibit broadly comparable improvements in total and regional adiposity.

A related implication of this study is that body weight alone is a weak indicator of intervention-related change in women with NWO. This phenotype is defined by a mismatch between a normal BMI and elevated adiposity; consequently, a stable body weight does not necessarily reflect an unchanged internal adiposity profile. This interpretation is consistent with previous studies showing that BMI-based categories can misclassify cardiometabolic health and that women with a normal BMI but elevated body fat may exhibit adverse metabolic features [4, 8–10, 12]. In the present study, women with NWO showed reductions across several adiposity-related outcomes despite minimal change in body weight, supporting the use of body composition and regional adiposity measures when evaluating intervention responses in this phenotype. Lean mass showed a small upward trend in the NWO group; however, this finding should be interpreted with caution and not overemphasized as definitive evidence of lean-mass gain. The reductions in VAT and android fat are clinically relevant, given that central adiposity is more strongly linked to cardiometabolic dysfunction than body weight per se. Previous studies have reported that women with NWO exhibit unfavorable metabolic and inflammatory profiles despite a normal BMI [9, 12], and that NWO is associated with cardiometabolic dysregulation and an elevated risk of cardiovascular mortality [8]. In the present study, both NWO and OB groups showed reductions in VAT and android fat mass over the 12-week intervention. This is noteworthy because NWO is frequently overlooked when BMI is used as the primary screening tool. The findings suggest that direct measurement of regional adiposity may reveal meaningful changes in women with NWO that would otherwise be missed by weight-centered evaluation. Nevertheless, in the absence of a non-exercise control group, the extent to which the observed changes can be specifically attributed to the exercise program cannot be determined.

Previous exercise intervention studies provide useful context for interpreting the present findings. Ho et al. reported that 12 weeks of aerobic, resistance, and combined exercise training elicited distinct patterns of change in fat mass, body weight, and cardiorespiratory fitness in adults with overweight or obesity [26]. Seo et al. showed that 12 weeks of combined exercise training changed body composition and metabolic syndrome-related factors in obese middle-aged women [27]. Sijie et al. reported that high-intensity interval training was associated with favorable changes in overweight young women [14]. More recent experimental work in obese women also supports the relevance of combined aerobic and resistance training for body composition-related outcomes [15]. Together with the present results, these studies suggest that mixed exercise programs may be effective in eliciting body composition and cardiorespiratory fitness adaptations, although the magnitude and pattern of change may differ across populations and outcome measures.

Routine cardiometabolic biomarkers showed a more limited pattern of change. LDL-C decreased in both groups at the nominal level, but the evidence was stronger in the OB group than in the NWO group after adjustment for multiple comparisons. Other lipid markers, including triglycerides, total cholesterol, and HDL-C, changed little. Similarly, fasting glucose, HbA1c, fasting insulin, and HOMA-IR did not show consistent changes. This pattern does not necessarily diminish the body composition findings; rather, it suggests that fasting blood biomarkers may be less sensitive than DXA-derived adiposity and fitness measures over a 12-week period, particularly when baseline glycemic and lipid values are close to the normal range. Hall et al. showed that continuous glucose monitoring (CGM) can reveal patterns of glucose dysregulation that are not captured by conventional fasting measures [28]. Future studies incorporating dietary monitoring and CGM may therefore provide a more comprehensive view of metabolic responses to exercise.

In contrast to the limited changes in fasting biomarkers, cardiorespiratory fitness provided a clearer functional signal. VO_2_max improved over time when expressed relative to body mass, in absolute terms, and relative to lean body mass. Reporting VO_2_max in these three forms is informative because the NWO and OB groups differ in body weight and lean mass. Mass-relative VO_2_max is clinically familiar but is influenced by body weight; absolute VO_2_max reflects total oxygen uptake capacity; and lean-mass-normalized VO_2_max contextualizes aerobic capacity with respect to metabolically active tissue. In the present study, the direction of improvement was consistent across all three expressions, adding an important functional dimension to the body composition findings. The clinical relevance of cardiorespiratory fitness is supported by large cohort studies showing that physical fitness and exercise capacity are strongly associated with long-term mortality risk [29–31].

Taken together, the present findings suggest that early changes during a supervised mixed-exercise program may be more readily detectable in body composition, regional adiposity, and cardiorespiratory fitness than in routine fasting metabolic biomarkers. This interpretation is particularly relevant for women with NWO. In this phenotype, the main clinical concern is not excess body weight per se, but excess adiposity concealed beneath a normal BMI. Accordingly, intervention evaluation should not rely primarily on body weight or BMI. A more appropriate assessment framework may combine DXA-derived regional adiposity outcomes with functional indicators such as VO_2_max. This approach better reflects the biological profile of NWO and may help prevent the underestimation of meaningful changes in women whose body weight remains within the normal range.

The strengths of this study include its phenotype-stratified design, the use of DXA-derived total and regional body composition outcomes, and the reporting of VO_2_max in multiple forms. These features enabled the detection of adiposity-related and functional changes that would not be adequately reflected by BMI or body weight alone. The study also contributes to the literature by comparing women with NWO and women with OB under the same supervised mixed-exercise program, using a consistent outcome framework that included body composition, regional adiposity, cardiometabolic biomarkers, and cardiorespiratory fitness.

Several limitations should be acknowledged. First, this study used a single-arm pre-post design without a non-exercise control group; therefore, the observed changes from baseline to week 12 cannot be definitively attributed to the supervised mixed-exercise program alone. Potential influences of time, measurement variability, regression to the mean, behavioral changes outside the intervention, and other unmeasured factors cannot be excluded. Future randomized controlled trials with non-exercise control groups are needed to confirm causal effects. Second, the final analysis was conducted on a per-protocol basis. Although participant retention and baseline characteristics were compared between completers and non-completers, residual attrition-related imbalance cannot be fully excluded. Third, no formal dietary intervention or precise dietary monitoring was implemented, which limits interpretation of changes in body composition and cardiometabolic biomarkers in relation to energy balance. Fourth, menstrual cycle phase, menopausal transition status, and hormonal contraceptive use were not systematically controlled. These factors may influence substrate metabolism, insulin sensitivity, lipid metabolism, fluid balance, and body composition estimates, thereby introducing additional variability into metabolic and body composition outcomes [32]. Finally, although the threshold of percent body fat > 30% for defining NWO in women is commonly applied, it is not universally accepted. Future studies should examine whether the present findings are reproducible when alternative sex, age, or ethnicity-specific body fat thresholds are applied.

## Conclusion

In this cohort-specific analysis, a 12-week supervised mixed-exercise program led to improvements in DXA-derived adiposity and cardiorespiratory fitness, while changes in fasting cardiometabolic biomarkers were modest and inconsistent. Similar patterns were observed in both women with NWO and OB, with no clear evidence of phenotype-specific differences.

Notably, women with NWO showed reductions in total and regional fat despite minimal changes in body weight, highlighting the limitations of BMI in capturing adiposity changes. However, these findings reflect within-cohort improvements rather than differential responses between phenotypes, as group × time interactions were not significant and baseline-adjusted differences were largely attenuated.

Overall, the results underscore the importance of assessing body composition and regional adiposity alongside traditional measures. Given the single-arm design, findings should be interpreted cautiously, and randomized controlled trials are needed to confirm causal effects.

## Supplementary Information

Supplementary Material 1.

Supplementary Material 2.

Supplementary Material 3.

Supplementary Material 4.

## Data Availability

The datasets generated and/or analysed during the current study are not publicly available due to the sensitive nature of the health data collected from the participants (obese female subjects) and the restrictions imposed by the Ethics Committee to protect participant privacy. Data are available from the corresponding author on reasonable request and with permission of the Ethics Committee.

## Abbreviations

1RM: One-repetition Maximum
ACSM: American College of Sports Medicine
A/G ratio: Android-to-gynoid ratio
BF%: Body Fat Percentage
BMI: Body Mass Index
CI: Confidence Interval
DBP: Diastolic Blood Pressure
DXA: Dual-energy X-ray Absorptiometry
HbA1c: Hemoglobin A1c
HDL-C: High-density Lipoprotein Cholesterol
LDL-C: Low-density Lipoprotein Cholesterol
HIIT: High-intensity Interval Training
HOMA-IR: Homeostatic Model Assessment for Insulin Resistance
HRR: Heart Rate Reserve
MD: Mean Difference
NWO: Normal-weight Obesity
OB: Obesity
RPE: Rating of Perceived Exertion
SD: Standard Deviation
SBP: Systolic Blood Pressure
TC: Total Cholesterol
TG: Triglycerides
VAT: Visceral Adipose Tissue
VO_2_max: Maximal Oxygen Uptake

## References

[1] Afshin A, Forouzanfar MH, Reitsma MB, Sur P, Estep K, Lee A, Marczak L, Mokdad AH, Moradi-Lakeh M, Naghavi M et al. Health Effects of Overweight and Obesity in 195 Countries over 25 Years. N Engl J Med 2017, 377(1):13–27. 10.1056/NEJMoa1614362 PMC5477817 28604169

[2] Blüher M. Obesity: global epidemiology and pathogenesis. Nat reviews Endocrinol. 2019;15(5):288–98. 10.1038/s41574-019-0176-8 30814686

[3] Nuttall FQ. Body Mass Index: Obesity, BMI, and Health: A Critical Review. Nutr Today. 2015;50(3):117–28. 27340299 10.1097/NT.0000000000000092 PMC4890841

[4] Tomiyama AJ, Hunger JM, Nguyen-Cuu J, Wells C. Misclassification of cardiometabolic health when using body mass index categories in NHANES 2005–2012. Int J Obes. 2016;40(5):883–6. 10.1038/ijo.2016.17 26841729

[5] Neeland IJ, Ross R, Després JP, Matsuzawa Y, Yamashita S, Shai I, Seidell J, Magni P, Santos RD, Arsenault B, et al. Visceral and ectopic fat, atherosclerosis, and cardiometabolic disease: a position statement. lancet Diabetes Endocrinol. 2019;7(9):715–25. 31301983 10.1016/S2213-8587(19)30084-1

[6] Tchernof A, Després JP. Pathophysiology of human visceral obesity: an update. Physiol Rev. 2013;93(1):359–404. 23303913 10.1152/physrev.00033.2011

[7] Zorena K, Jachimowicz-Duda O, Ślęzak D, Robakowska M, Mrugacz M. Adipokines and Obesity. Potential link to metabolic disorders and chronic complications. Int J Mol Sci. 2020;21(10):3570. 10.3390/ijms21103570 PMC7278967 32443588

[8] Romero-Corral A, Somers VK, Sierra-Johnson J, Korenfeld Y, Boarin S, Korinek J, Jensen MD, Parati G, Lopez-Jimenez F. Normal weight obesity: a risk factor for cardiometabolic dysregulation and cardiovascular mortality. Eur Heart J. 2010;31(6):737–46. 19933515 10.1093/eurheartj/ehp487 PMC2838679

[9] De Lorenzo A, Martinoli R, Vaia F, Di Renzo L. Normal weight obese (NWO) women: an evaluation of a candidate new syndrome. Nutr metabolism Cardiovasc diseases: NMCD. 2006;16(8):513–23. 17126766 10.1016/j.numecd.2005.10.010

[10] Correa-Rodríguez M, González-Ruíz K, Rincón-Pabón D, Izquierdo M, García-Hermoso A, Agostinis-Sobrinho C, Sánchez-Capacho, N, Roa-Cubaque MA, Ramírez-Vélez, R. Normal-weight obesity is associated with increased cardiometabolic risk in young adults. Nutrients. 2020;12(4):1106. 10.3390/nu12041106 PMC7230158 32316150

[11] Mohammadian Khonsari N, Khashayar P, Shahrestanaki E, Kelishadi R, Mohammadpoor Nami S, Heidari-Beni M, Esmaeili Abdar Z, Tabatabaei-Malazy O, Qorbani M. Normal Weight Obesity and Cardiometabolic Risk Factors: A Systematic Review and Meta-Analysis. Front Endocrinol. 2022;13:857930. 10.3389/fendo.2022.857930 PMC8987277 35399938

[12] De Lorenzo A, Del Gobbo V, Premrov MG, Bigioni M, Galvano F, Di Renzo L. Normal-weight obese syndrome: early inflammation? Am J Clin Nutr. 2007;85(1):40–5. 17209175 10.1093/ajcn/85.1.40

[13] Ahmed KY, Aychiluhm SB, Thapa S, Tegegne TK, Ketema DB, Kassa ZY, Kibret GD, Duko B, Shifti DM, Bore MG, et al. Cardiometabolic Outcomes Among Adults With Abdominal Obesity and Normal Body Mass Index. JAMA Netw Open. 2025;8(10):e2537942. 41105408 10.1001/jamanetworkopen.2025.37942 PMC12534855

[14] Sijie T, Hainai Y, Fengying Y, Jianxiong W. High intensity interval exercise training in overweight young women. J Sports Med Phys Fit. 2012;52(3):255–62. 22648463

[15] Wen HJ, Liu SH, Tsai CL. Effects of 12 weeks of aerobic exercise combined with resistance training on neurocognitive performance in obese women. J Exerc Sci Fit. 2022;20(4):291–304. 35892114 10.1016/j.jesf.2022.07.001 PMC9287612

[16] Lemos T, Gallagher D. Current body composition measurement techniques. Curr Opin Endocrinol Diabetes Obes. 2017;24(5):310–4. 28696961 10.1097/MED.0000000000000360 PMC5771660

[17] Oppert JM, Bellicha A, van Baak MA, Battista F, Beaulieu K, Blundell JE, Carraça EV, Encantado J, Ermolao A, Pramono A, et al. Exercise training in the management of overweight and obesity in adults: Synthesis of the evidence and recommendations from the European Association for the Study of Obesity Physical Activity Working Group. Obes Rev. 2021;22(Suppl 4):e13273. 34076949 10.1111/obr.13273 PMC8365734

[18] Chen X, He H, Xie K, Zhang L, Cao C. Effects of various exercise types on visceral adipose tissue in individuals with overweight and obesity: A systematic review and network meta-analysis of 84 randomized controlled trials. Obes Rev. 2024;25(3):e13666. 38031812 10.1111/obr.13666

[19] Maillard F, Pereira B, Boisseau N. Effect of High-Intensity Interval Training on Total, Abdominal and Visceral Fat Mass: A Meta-Analysis. Sports Med (Auckland NZ). 2018;48(2):269–88. 10.1007/s40279-017-0807-y 29127602

[20] Lopez P, Taaffe DR, Galvão DA, Newton RU, Nonemacher ER, Wendt VM, Bassanesi RN, Turella DJP, Rech A. Resistance training effectiveness on body composition and body weight outcomes in individuals with overweight and obesity across the lifespan: A systematic review and meta-analysis. Obes Rev. 2022;23(5):e13428. 35191588 10.1111/obr.13428 PMC9285060

[21] Cao J, Lei S, Zhao T, Xie Y, Zhou Z, Cheng S, Wang X. Changes in fat oxidation and body composition after combined exercise intervention in sedentary obese Chinese adults. J Clin Med. 2022;11(4):1086. 10.3390/jcm11041086 PMC8879656 35207356

[22] Zhang B, Yang Z, Yin M, Xie Y, Zuo H, Zhang T, Lei S, Wang C, Cheng S, Wang X. Effectiveness of combined exercise intervention on sedentary behaviour patterns, body composition, and cardiometabolic health among middle-aged adults. BMC sports Sci Med rehabilitation. 2025;18(1):45. 10.1186/s13102-025-01488-6 PMC12859953 41456073

[23] China WGoOi. Guidelines for prevention and control of overweight and obesity in Chinese adults. Acta nutrimenta sinica. 2004;26(1):1–4.

[24] Zhou B-F. Predictive values of body mass index and waist circumference for risk factors of certain related diseases in Chinese adults–study on optimal cut-off points of body mass index and waist circumference in Chinese adults. Biomed Environ Sci: BES. 2002;15(1):83–96. 12046553

[25] Thompson PD, Arena R, Riebe D, Pescatello LS. ACSM’s new preparticipation health screening recommendations from ACSM’s guidelines for exercise testing and prescription, ninth edition. Curr Sports Med Rep. 2013;12(4):215–7. 23851406 10.1249/JSR.0b013e31829a68cf

[26] Ho SS, Dhaliwal SS, Hills AP, Pal S. The effect of 12 weeks of aerobic, resistance or combination exercise training on cardiovascular risk factors in the overweight and obese in a randomized trial. BMC Public Health. 2012;12:704. 23006411 10.1186/1471-2458-12-704 PMC3487794

[27] Seo DI, So WY, Ha S, Yoo EJ, Kim D, Singh H, Fahs CA, Rossow L, Bemben DA, Bemben MG, et al. Effects of 12 weeks of combined exercise training on visfatin and metabolic syndrome factors in obese middle-aged women. J sports Sci Med. 2011;10(1):222–6. 24149317 PMC3737906

[28] Hall H, Perelman D, Breschi A, Limcaoco P, Kellogg R, McLaughlin T, Snyder M. Glucotypes reveal new patterns of glucose dysregulation. PLoS Biol. 2018;16(7):e2005143. 30040822 10.1371/journal.pbio.2005143 PMC6057684

[29] Blair SN, Kohl HW 3rd, Paffenbarger RS Jr., Clark DG, Cooper KH, Gibbons LW. Physical fitness and all-cause mortality. A prospective study of healthy men and women. JAMA. 1989;262(17):2395–401. 2795824 10.1001/jama.262.17.2395

[30] Myers J, Prakash M, Froelicher V, Do D, Partington S, Atwood JE. Exercise capacity and mortality among men referred for exercise testing. N Engl J Med. 2002;346(11):793–801. 11893790 10.1056/NEJMoa011858

[31] Mandsager K, Harb S, Cremer P, Phelan D, Nissen SE, Jaber W. Association of Cardiorespiratory Fitness With Long-term Mortality Among Adults Undergoing Exercise Treadmill Testing. JAMA Netw Open. 2018;1(6):e183605. 30646252 10.1001/jamanetworkopen.2018.3605 PMC6324439

[32] Elliott-Sale KJ, Minahan CL, de Jonge X, Ackerman KE, Sipilä S, Constantini NW, Lebrun CM, Hackney AC. Methodological Considerations for Studies in Sport and Exercise Science with Women as Participants: A Working Guide for Standards of Practice for Research on Women. Sports Med (Auckland NZ). 2021;51(5):843–61. 10.1007/s40279-021-01435-8 PMC8053180 33725341

