# Supplementary material for: Changes in visceral adiposity, body composition, and cardiometabolic profiles following a supervised mixed exercise program in women with normal-weight obesity and obesity
Source: BMC Sports Sci Med Rehabil. 2026 Jun 11;18:359. doi: 10.1186/s13102-026-01796-5 (PMC13480087; doi:10.1186/s13102-026-01796-5)
Supplement: Supplementary file 1 [file 13102_2026_1796_MOESM1_ESM.pdf]

International Physical Activity Questionnaire (IPAQ)

About: This scale is a self-report measure of physical activity.

Items: 12

Reliability:
Test-retest reliability ranged between 0.96 and 0.46, with an average of about 0.8.

Validity:
Correlations between this self-report sleep scale and the MOS Sleep subscales were statistically significant with the exception of the MOS Snoring subscale.

Scoring:
There was fair to moderate agreement between the IPAQ and accelerometer-measured physical activity and sitting time.

References:
Craig, C. L., Marshall, A. L., Sjöström, M., Bauman, A. E., Booth, M. L., Ainsworth, B. E., Pratt, M., Ekelund, U., Yngve, A., Sallis, J. F., Oja, P. (2003). International Physical Activity Questionnaire: 12-country reliability and validity. *Medicine and Science in Sports and Exercise, 35*(8), 1381-1395.

International Physical Activity Questionnaire (IPAQ)

The following questions are about physical activity. Please read carefully and answer to the best of your knowledge.

1. When you are at work, which of the following describes what you do? (If you have more than one job, answer for each job.)

Job 1	Job 2	Job 3
☐ Mostly sitting or standing	☐ Mostly sitting or standing	☐ Mostly sitting or standing
☐ Mostly walking	☐ Mostly walking	☐ Mostly walking
☐ Mostly heavy labor or physically demanding work	☐ Mostly heavy labor or physically demanding work	☐ Mostly heavy labor or physically demanding work
☐ Don't know/not sure	☐ Don't know/not sure	☐ Don't know/not sure

The next 3 questions are about vigorous physical activity. Vigorous physical activity causes your heart to beat faster and makes you breathe hard.

2. Now thinking about **vigorous physical activities** you did in the **last 7 days**, did you do vigorous activities for at least 10 minutes at a time, such as running, aerobics, sports, heavy yard work, or anything else?

☐ 1 Yes

☐ 0 No→ **Skip to 15 on the next page →**

3. How many days per week do you do these vigorous activities for at least 10 minutes at a time?

Days per week: 1 2 3 4 5 6 7

4. On the days when you do vigorous activity for at least 10 minutes, how long do you do those activities?

☐ 1 About 10 minutes

☐ 2 About 20 minutes

☐ 3 About 30 minutes

☐ 4 About 40 minutes

☐ 5 About 50 minutes

☐ 6 1 hour or more

The next 3 questions are about moderate physical activity. Moderate physical activity causes small increases in breathing or heart rate.

5. Now thinking about **moderate physical activities** you did in the **last 7 days**, did you do moderate activities for at least 10 minutes at a time, such as brisk walking, bicycling at regular pace, vacuuming, gardening, or anything else?

☐ 1 Yes

☐ 0 No→ **Skip to 18 on the next page →**

6. How many days per week do you do these moderate activities for at least 10 minutes at a time?

Days per week: 1 2 3 4 5 6 7

7. On the days when you do moderate activity for at least 10 minutes, how long do you do those activities?

☐ 1 About 10 minutes
☐ 2 About 20 minutes
☐ 3 About 30 minutes
☐ 4 About 40 minutes
☐ 5 About 50 minutes
☐ 6 1 hour or more

The next 3 questions are about time spent walking.

8. Now thinking about the amount of time you spent **walking** in the **last 7 days**, that lasted for at least 10 minutes at a time. This includes at work and at home, walking to travel from place to place, and any other walking that you might do solely for recreation, sport, exercise, or leisure.

☐ 1 Yes

☐ 0 No→ **Skip to 22 on the next page →**

9. How many days per week do you do spend walking for at least 10 minutes at a time?

Days per week: 1 2 3 4 5 6 7

10. On the days when you do walk for at least 10 minutes, how long do you do those activities?

- ☐ 1 About 10 minutes
- ☐ 2 About 20 minutes
- ☐ 3 About 30 minutes
- ☐ 4 About 40 minutes
- ☐ 5 About 50 minutes
- ☐ 6 1 hour or more

The next 3 questions are about you sitting.

11. Now thinking about the amount of time you spent **sitting** in the **last 7 days.** Include time spent at work, at home, while doing course work and during leisure time. This may include time spent sitting at a desk, visiting friends, reading, or sitting or lying down to watch television.

12. How many hours per day do you spend sitting?

- ☐ 1 About 30 minutes or less
- ☐ 2 About 1 hour
- ☐ 3 About 2 hour
- ☐ 4 About 3 hour
- ☐ 5 About 4 hour
- ☐ 6 About 5 hour or more
